# The relationship between vitamin D status, intake and exercise performance in UK University-level athletes and healthy inactive controls

**DOI:** 10.1371/journal.pone.0249671

**Published:** 2021-04-02

**Authors:** Saskia L. Wilson-Barnes, Julie E. A. Hunt, Jeewaka Mendis, Emma L. Williams, David King, Harry Roberts, Susan A. Lanham-New, Ralph J. F. Manders

**Affiliations:** 1 Department of Nutritional Sciences, School of Biosciences and Medicine, Faculty of Health and Medical Sciences, University of Surrey, Guildford, Surrey, United Kingdom; 2 Surrey Clinical Research Centre, Surrey Clinical Trials Unit, NIHR Research Design Service SE, Guildford, Surrey, United Kingdom; 3 Division of Women’s, Children’s and Clinical Support, Imperial College Healthcare NHS Trust, London, United Kingdom; Universitat de les Illes Balears, SPAIN

## Abstract

The potential ergogenic effects of vitamin D (vitD) in high performing athletes has received considerable attention in the literature and media. However, little is known about non-supplemented university athletes and students residing at a higher latitude. This study aimed to investigate the effects of vitD (biochemical status and dietary intake) on exercise performance in UK university athletes and sedentary students. A total of 34 athletes and 16 sedentary controls were studied during the spring and summer months. Serum vitD status and sunlight exposure were assessed using LC-MS/MS and dosimetry, respectively. Muscular strength of the upper and lower body was assessed using handgrip and knee extensor dynamometry (KE). Countermovement jump (CMJ) and aerobic fitness were measured using an Optojump and VO_2max_ test, respectively. Statistical analysis was performed using paired/ independent t-tests, ANCOVA and Pearson/ Spearman correlations, depending on normality. VitD status increased significantly over the seasons, with athletes measuring higher status both in spring (51.7±20.5 vs. 37.2±18.9 nmol/L, p = 0.03) and summer (66.7±15.8 vs 55.6±18.8 nmol/L, p = 0.04) when compared to controls, respectively. Notably, 22% of the subjects recruited were vitD deficient during the spring term only (<25nmol/L, *n* 9). Subjects with ‘insufficient’ vitD status (<50nmol/L) elicited significantly lower CMJ when contrasted to the vitD ‘sufficient’ (>50nmol/l) group (p = 0.055) and a lower VO_2 max_ (p = 0.05) in the spring and summer term (p = 0.05 and p = 0.01, respectively). However, an ANCOVA test showed no significant difference detected for either CMJ or VO_2max_ following adjustments for co-variates. In conclusion, we provide novel information on the vitD status, dietary intake, physical fitness and sunlight exposure of UK young adults across two separate seasons, for which there is limited data at present.

## Introduction

Research in vitamin D has increased in recent years and is attributed to its multiple roles in human health, including the effects on musculoskeletal, cardiovascular health, and immune function [1]. Correspondingly, there is a growing interest within the sport science field suggesting that increasing vitamin D status could play a role in optimising sport performance [2]. However, due to the large heterogeneity in research studies, the effects of vitamin D status on sport performance remains to be fully defined.

Vitamin D is a unique pro-hormone and is not considered a “vital amine”, as our primary source is through subcutaneous exposure to ultraviolet B radiation (UVB). Following exposure, a chain reaction within the skin forms cholecalciferol (or vitamin D_3_), which is thereby further hydroxylated to form the circulating metabolite 25-hydroxyvitamin D (25(OH)D). This is most commonly measured in vitamin D research [1] due to its stability and relatively long half-life (28 days). Variables which may negatively influence vitamin D status within healthy individuals include ethnicity, seasonality and geographical location [3]. When the latter was evaluated in a cross-site study at 3 different latitudes, the zenith angle of the sun was shown to increase photosynthesis of pre-vitamin D_3_ in the spring and summer months [3]. Within the UK, the production of pre-vitamin D_3_ only occurs between April and September from 11am-3pm due to a latitude greater than 40°N of the equator [3, 4]. Therefore, those that spend the majority of their day indoors are susceptible to year-round vitamin D insufficiency (deficiency can be defined as a vitamin D status of <25nmol/L within the UK [5]; sufficiency is considered to be >50nmol/L [6]).

Vitamin D sources within the diet are limited, particularly foods that have a large vitamin D availability on average per serving [5]. Other countries, such as Finland, have substantially improved their public health by correcting 25(OH)D status through the successful implementation of a food fortification policy [7]. Vegetarians and vegans are also at considerable risk of not meeting their recommended nutrient intake (RNI) as key vitamin D food sources are derived from animal products and meats [5]. This is further confounded by the fact that vegans may potentially not want to consume vitamin D_3_ supplements as these are often synthesized by the UVB irradiation of lanolin (sheep’s wool) by most companies [5]. Although there have been recent advances regarding the requirements for vitamin D within the UK following the Scientific Advisory Committee on Nutrition (SACN) updating and increasing the RNI for adults (19–64 years) from 0 to 10 μg/d [5]. There is currently limited data for vitamin D intake within young adult population groups, such as university students.

Due to its influences upon calcium homeostasis, the specific effects of vitamin D may exert indirect effects on muscular function [8–10]. Within professional athletic populations, there is conflicting evidence for the effects of a ‘sufficient’ vitamin D status on predictors of upper- and lower body strength and power [1, 2, 10, 11], which is attributable to the heterogeneity across studies with regards to diverse populations, ethnicities, geographical location and types of sport performance measurements [11]. Such as, a study investigating US University-level taekwondo athletes (30.4°N) found that they had improved their wingate anaerobic fitness from 10.4 to 11.6 W/kg following the correction of their vitamin D status [12]. However, a training programme was administered to the control and intervention group thus, it is unclear whether the improvement in aerobic capacity was exclusively due to the improvement in vitamin D status. Furthermore, there is limited research amongst non-professional athletes (such as university-level athletes) residing within the UK and considering there are 170 institutions hosting weekly British Universities and Colleges Sport (BUCS) fixtures across 50 different disciplines receiving little nutritional support [13] more research in this field is warranted. In addition to this, vitamin D status in young free-living adults is rarely explored, specifically for sedentary University students. This is because the vast majority of vitamin D research focusses upon children/adolescent or elderly and institutionalised populations due to its direct influence on bone.

Research amongst vitamin D ‘deficient’ (<30 nmol/L) Sprague-Dawley rats exhibit a higher contractile response of the isolated cardiac and vascular smooth muscle [14], which is attributed to the presence of the VDR within the cardiovascular system. Therefore, it is proposed that vitamin D, specifically the active form; 1,25 (OH)_2_D, may influence aerobic capacity and fitness [15]. Although the mechanisms remain unclear, recent studies observed associations between vitamin D and aerobic fitness. In an observational study on British army recruits (53°N) it was found that a sufficient vitamin D status (>50nmol/L) was associated with faster 1.5 mile run times [16]. A cross-sectional study from the US (30.4°N) examining recreational athletes also suggested a positive association between aerobic capacity and vitamin D sufficiency (≤87.5nmol/L) [17]. Therefore, further research is warranted to examine whether an improvement in vitamin D status has an effect upon aerobic fitness.

The primary aim of this study was to determine the relationship between vitamin D status and exercise performance in young active adults. The secondary aim was to provide information on the vitamin D intake of healthy UK-dwelling young adults. We hypothesized that athletes and controls alike will have an insufficient (< 50nmol/L) vitamin D status during the spring thereby adversely affecting exercise performance. During the summer period, we anticipated that the vitamin D status will improve for both the athletes and controls.

## Methods

### Subjects & protocol

Physically healthy male and female university students and athletes from the University of Surrey (51.2°N) were recruited between January and March (2018) to take part in this study. Participants were recruited utilising posters and leaflets placed in communal areas and social media sites (with permission from administrators) around the University of Surrey. Coaching staff and signatories of sports teams were also approached by the research team to attend training sessions. Athletes were considered eligible if they were a competitive member of a university sports team, white, aged 18–30 years and had a BMI between 18 and 30 kg/m^2^. Healthy inactive controls were included if they were aged 18–30 years, BMI (18–30 kg/m^2^), white and did not exercise for more than 150 minutes/week. Potential participants were excluded if they used sun beds, vitamin D supplements or were planning a sun holiday during the study (February-June 2018). All participants were required to complete a health screening questionnaire to control for medical conditions/medication use that were likely to affect vitamin D metabolism [18]. A total of 50 participants (n = 24 males, n = 26 females) were included, 34 (n = 18 male, n = 16 female) were university athletes competing in a variety of sports such as, but not limited to: rowing, basketball, cycling, racket sports, rugby, swimming or triathlon. The remaining 16 participants were recruited as controls (n = 6 male, n = 10 female).

This longitudinal study assessed vitamin D status, dietary intake and exercise performance from spring (February/March 2018) to summer (May/June 2018) over the course of a competitive sporting season (BUCS) at the University of Surrey. Ethical approval for the study was provided by the National Health Research Authority (HRA); REC reference 17/LO/1699.

**Fig 1:**
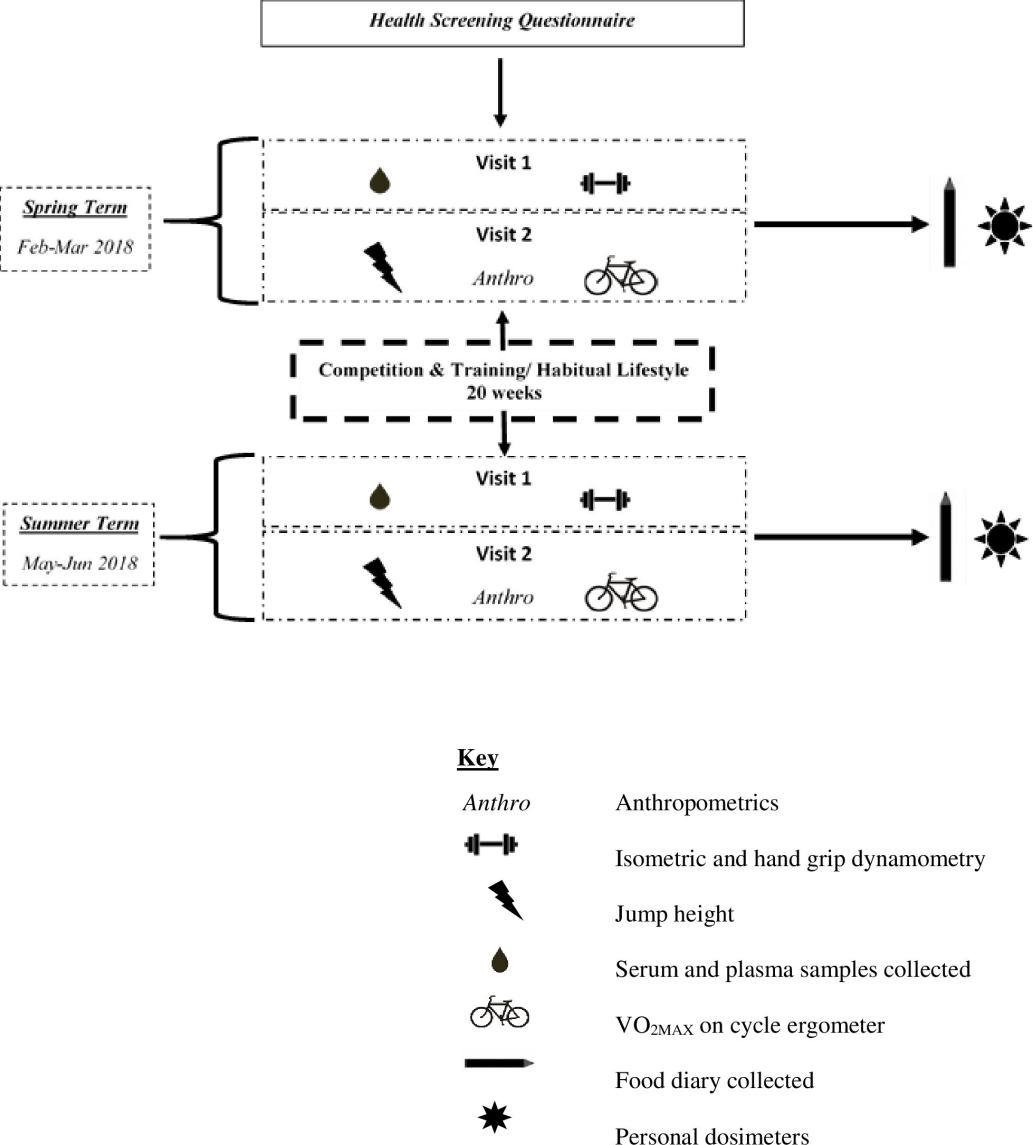
Schematic diagram of protocol.

Test periods consisted of two separate study days, which were performed in the same order at baseline (spring) and the second visit (summer). On day one, participants provided written informed consent, after which they provided a fasted blood sample and conducted upper and lower body muscular strength assessments, as shown in Fig 1. On the second day, participants attended the laboratory and completed an assessment of CMJ height and aerobic fitness. Body composition was measured through the use of a dual-energy x-ray absorptiometry (DEXA) whole body scan (Hologic QDR, Hologic inc. USA). Participants were instructed to refrain from any strenuous or moderate exercise 24 hours before the scans and blood samples, in light of the evidence that vitamin D may mobilise from adipose tissue following exercise [19]. Participants were encouraged to wear the same items of light clothing at both time points. Self-reported 5-day food diaries were collected to assess average calcium and vitamin D intake. Sunlight exposure was measured through the use of UVB dosimetry using badges composed of a polysulphone film which were provided on day 2. For the measurement of UV exposure, the badges were read at 330nm on a spectrophotometer (Thermo- Scientific Evolution, Fisher Scientific) prior to and after use. The participants were instructed to wear these personal dosimeter badges on their outdoor clothing for 5 days. To detect the amount of UV light the participant would habitually be subjected to during their daily routine the badges were thereby translated to standard erythemal dose (SED) measurements using the following mathematical formula:

$$SED=10.7\;{\left[\Delta A_{330}]+14.3\;[\Delta A_{330}\right]}^{2}–26.4\;{\left[\Delta A_{330}\right]}^{3}+89.1\;{\left[\Delta A_{330}\right]}^{4}$$

Where ΔA_330_ is the change in the absorbance of the film badge from pre-to post-UVB exposure [20]. 1 SED is equivalent to 100 J m^2^ of erythemal (sun burning) UV radiation and is generally used as a measure of UV exposure [21].

For blood collection, participants were instructed to visit the lab after an overnight fast (≥8 hours). Blood was collected from the anti-cubital vein, plasma (6 mL) in EDTA containing tubes and centrifuged at 1,300 *g* and 4°C for 10 min and serum (10mL) was kept at room temperature to clot for an hour before centrifugation at 1,300 *g* and 22°C. Aliquots of plasma and serum were frozen and stored at -20°C until analyses using liquid chromatograph-tandem mass spectrometry (LC-MS/MS) to measure 25(OH)D using a Waters Acuity TQD using PFP column following supported liquid extraction (SLE).

### Exercise performance

Muscle strength of the upper body was assessed using a hand grip dynamometer (5401 Takei Scientific Instruments Co. Japan). Participants were instructed to hold the dynamometer in their dominant hand whilst standing and to hold the instrument above their heads and squeeze whilst returning their arm to their hip with fully extended elbow. This was repeated three times consecutively, the highest reading from the attempts has been reported (kg).

Participants were asked to complete 3 countermovement jumps without arm swing separated by a minute’s rest using an Optojump (Microgate Co., NY). Participants were instructed to complete a vertical jump for familiarisation and to ensure the correct technique was performed. Jump technique was from a standing position with hands on hips.

Isometric torque of the knee extensor muscles was assessed on an isokinetic dynamometer (CSMI Humac Norm, Stoughton, MA). Participants completed a 5-min warm up on a cycle ergometer (~75W) before being seated on the isokinetic dynamometer with their non-dominant leg secured at 90^o^ knee flexion. The non-dominant leg was identified by the participant as the leg they do not strike off first when walking from a standstill position. Participants were instructed to perform 3 sub-maximal contractions (25, 50 and 75% of maximal load) separated by a 30 second rest to familiarise them with the equipment. Peak isometric knee extensor torque (KET) was measured through 3, 5-second maximal contractions separated by 1-minute rest. KET was determined from the highest torque measurement (Nm) of all 3 maximal contractions is reported.

Aerobic fitness was tested using a VO_2max_ exercise protocol using a stationary cycle ergometer (Monark LC6 Novo, Monark Sweden), the test consisted of progressive increments in cycling workload until volitional fatigue. Athletes warmed up at a set power output of 100W for females and 125W males, controls warmed up at 75W for females and 100W for males for 3 minutes before commencing a ramp test with an increment of 25W/minute at a cadence of >70 rpm. VO_2max_ was determined using a Vyntus CPX (Vyaire medical inc.) metabolic cart. All participants wore a heart rate strap to monitor and measure maximal heart rate (Polar T31, Polar Electro). Once participants reached maximal exertion or could not maintain a cadence above 60rpm despite verbal encouragement the test was stopped immediately, and they were instructed to continue cycling for a cool down of 3 minutes at 75W.

### Statistical analysis

All statistical analysis was performed using SPSS (Version 24; SPSS Inc., Chicago, 2018). Data were checked for normality using Shapiro-Wilk’s. Paired and independent t-tests or the non-parametric equivalents were carried out on this dataset. Correlations were examined using Pearson or Spearman (controlling for lean body mass, height and weight), depending on data normality. A two-way between-groups analysis of variance (ANOVA) was also conducted to explore the impact of sport participation (controls vs university athletes) and vitamin D status (sufficiency vs insufficiency) upon predictors of exercise performance during both seasons. An ANCOVA was run to assess the impact of a number of variables upon exercise performance, including gender, sport participation, lean body mass (LBM), body fat (BF) and age. Significance was set at p≤0.05 and all data were presented as mean±SD throughout.

## Results

### Subject characteristics

Athletes were younger (20.8 ± 1.9 vs. 24.8 ± 4.2 y, p<0.001) than the control population and both groups had a healthy BMI, on average (23.2 ± 2.3 vs 24.1 ± 4.2 kg/m^2^, respectively, p = 0.2, Table 1). Athletes exhibited a significantly lower body fat percentage when compared to their control counterparts in the spring (21.5 ± 6.9% vs. 28.2 ± 6.7%, p = 0.003) but not in the summer term (22.8 ± 7.7% vs 26.6 ± 7.4%, p = 0.126). There was no difference in BF between the control and athlete group following an ANCOVA test adjusting for gender (*F* (1,44) = 1.043, p = 0.3, partial η^2^ = .023). Lean body mass did not differ between the groups in spring (56.0 ± 11.2 vs. 49.8 ± 11.6 kg; p = 0.09) or summer (53.8 ± 10.5 vs. 50.8 ± 12.1, p = 0.4).

**Table 1:** Participant demographics and physical performance parameters.

	Spring			Summer		
	Athletes (n = 34)	Controls (n = 16)	Combined (n = 50)^1^	Athletes (n = 34)^2^	Controls (n = 16)^2^	Combined (n = 50) ^2^^,^^3^
**Age (y)**	20.8±1.9	24.8±4.2	22±3.3***	-	-	-
**Height (m)**	1.76±0.1	1.69±0.1	1.74±0.1*	-	-	-
**BMI (kg/m^2^)**	23.2±2.3	24.1±4.2	23.5±2.3	22.9±2.0	23.7±1.8	23.2±2.0
**Body Fat (%)**	21.5±6.9	28.2±6.7	23.6±7.5**	22.8±7.7	26.6±7.4^*^	24.1±7.7
**LBM (kg)**	56.0±11.2	49.8±11.6	54.1±11.5	53.7±10.5	50.8±12.0	52.8±11.0
**Serum 25(OH)D (nmol/L)**	51.7±20.5	37.2±18.9	46.7±20.9*	66.7±15.8^**α**^	55.6±18.8^*^	63.1±17.3**^α^
**Dietary vitamin D intake (μg/d)**	3.0±2.5	3.9±2.9	3.3±2.7	2.7±2.3	5.9±4.8	3.3±3.3^α^
**SED**	1.4±1.1	0.8±1.0	1.2 ±1.1	6.8±17.3	9.1±8.6^*^	7.4±15.2**
**Training (hr)**	6.8±4.6	N/A	N/A	-	-	-
**Handgrip (kg)**	39.2±8.7	31.1±9.6	37.2±9.5**	41.5±9.6^α^	36.8±14.9	39.9±11.7**
**KE (Nm)**	241.6±73.8	207.7±67.3	230.5±72.8	251.3±103.2	219.5±72.3	240.5±81.3
**CMJ (cm)**	35.7±6.8	26.6±8.4	31.5±8.7**	32.8±8.9	26.0±7.5	30.5±9.0^α^
**VO_2MAX_ (ml/kg/min^-1^)**	47.3±8.6	34.8±8.8	43.4±10.4***	47.8±10.6	35.8±5.1^*^	44.0±10.7^αα^
**HR_MAX_ (bpm)**	189.8±9.5	185.6±11.1	188.4±10.1	189.0±9.3	183.2±8.8	187.1±9.4

Values mean ± SD.

BMI: body mass index; LBM: lean body mass; 25(OH)D: serum 25-hydroxyvitamin D; Training: self-reported hours; SED: standard erythemal dose.

^1^ Independent t-test between athletes and controls during the spring season*<0.05 **<0.01 ***<0.001.

^2^ Paired t-test: ^α^ between athletes (p<0.05)

* between controls (p<0.05)

**between combined (p<0.05).

^3^ Independent t-test between athletes and controls during the summer season ^α^<0.05, ^αα^p<0.001.

### Vitamin D intake, status & sunlight exposure

**Fig 2:**
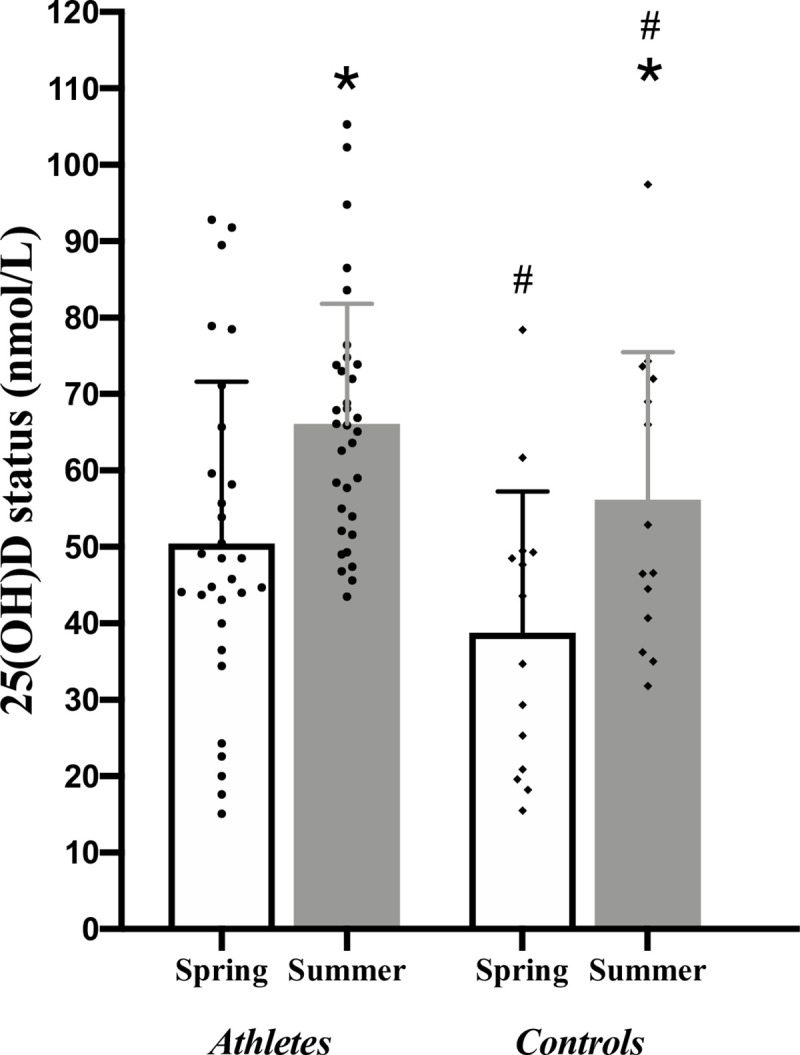
Seasonal variation in vitamin D status between the athlete and control group. *p<0.05 for the athletes/control groups between seasons. ^#^p<0.05 between athlete and control groups in spring and summer terms.

Vitamin D status increased significantly from 46.7 ± 20.9 in spring to 63.1 ± 17.3 nmol/L in summer (p<0.001) for the combined groups, as presented in Table 1. Vitamin D status was higher in athletes both in the spring (51.7 ± 20.5 vs. 37.2 ± 18.9 nmol/L, p = 0.03) and summer (66.7 ± 15.8 vs 55.6 ± 18.8 nmol/L, p = 0.04). The individual variation of vitamin D status between the groups throughout the seasons is presented in Fig 2. During the spring term 46% (n = 17) of the athletes had an insufficient vitamin D status (<50 nmol/L), insufficiency rates within the control cohort was 87.5% (n = 14). Notably, nine of the participants were considered to be vitamin D deficient ((< 25nmol/L) athletes n = 4; controls n = 5), In the summer term 26% (athletes: n = 5; controls: n = 8) of the cohort had an insufficient vitamin D status (<50 nmol/L) despite an increase in SED from 1.2 to 7.4 (equivalent to a change from 120 to 740 J/m^2^; p = 0.02) for both groups combined. During the summer term, none of the participants had vitamin D deficiencies.

Dietary analysis revealed that our cohort did not meet the current UK recommendations for vitamin D (10 μg/d, [5]). In the athlete group dietary analysis revealed an average intake of 3.0 ± 2.5 μg/d, whereas the control group reported an intake of 3.9 ± 2.9μg/d during the spring (p = 0.3). Dietary analysis during the summer term revealed that vitamin D intake did not change within the athlete group (2.7 ± 2.3 μg/d (p = 0.95)), whilst the control group were found to have a higher intake of 5.9 ± 4.8 μg/d (p = 0.62). Pearson correlations revealed that there was no association between vitamin D intake and status in the spring (r = -0.159; p = 0.401) or summer (r = -0.228; p = 0.242) when both groups were combined.

### Exercise performance

The athlete group were physically fitter as they were observed to present with a higher upper body muscular strength, CMJ height and aerobic fitness at baseline/spring than the controls (Table 1). Upper body strength was higher in the athlete group during the spring (39.2 ± 8.7 vs. 31.1 ± 9.6 kg, p = 0.01) only. There was no statistical difference detected between athletes and control group during each term for predictors of lower body muscle strength in spring (241.6 ± 73.8 vs. 207.7 ± 67.3 Nm, p = 0.1) and summer (251.3 ± 103.2 vs. 219.5 ± 72.3 Nm, p = 0.2). CMJ height was significantly higher in the athlete group during the spring (35.7 ± 6.8 vs. 26.6 ± 8.4 cm p = 0.008) and in the summer (32.8 ± 8.9 vs. 26.0 ± 7.5 cm, p = 0.02) when compared to the control group. Aerobic fitness was higher in the athletic group during the spring (47.3 ± 8.6 vs 34.8 ± 8.8 ml/kg/min^-1^, p = <0.001) and summer (47.8 ± 10.6 vs 35.8 ± 5.1 ml/kg/min^-1^, p<0.001) term. Following Pearson correlation testing, there was no association between vitamin D status and exercise performance in the sport, control or combined groups. This was also the case when controlling for lean body mass, height and weight.

Insufficient vitamin D levels were reported within 58% of the University-level athletes during the spring and 36% in the summer. When divided into sufficiency groups Pearson correlations only detected an association in spring between vitamin D status and CMJ height (r = 0.502, p = 0.02) but not for aerobic fitness (r = 0.279, p = 0.07) within the sufficient group. The interaction between vitamin D status and sport participation was not statistically significant for handgrip (p = 0.1), CMJ height (p = 0.2), aerobic fitness (p = 0.4) or KET (p = 0.3) during the summer following two-way between-groups ANOVA testing.

**Fig 3:**
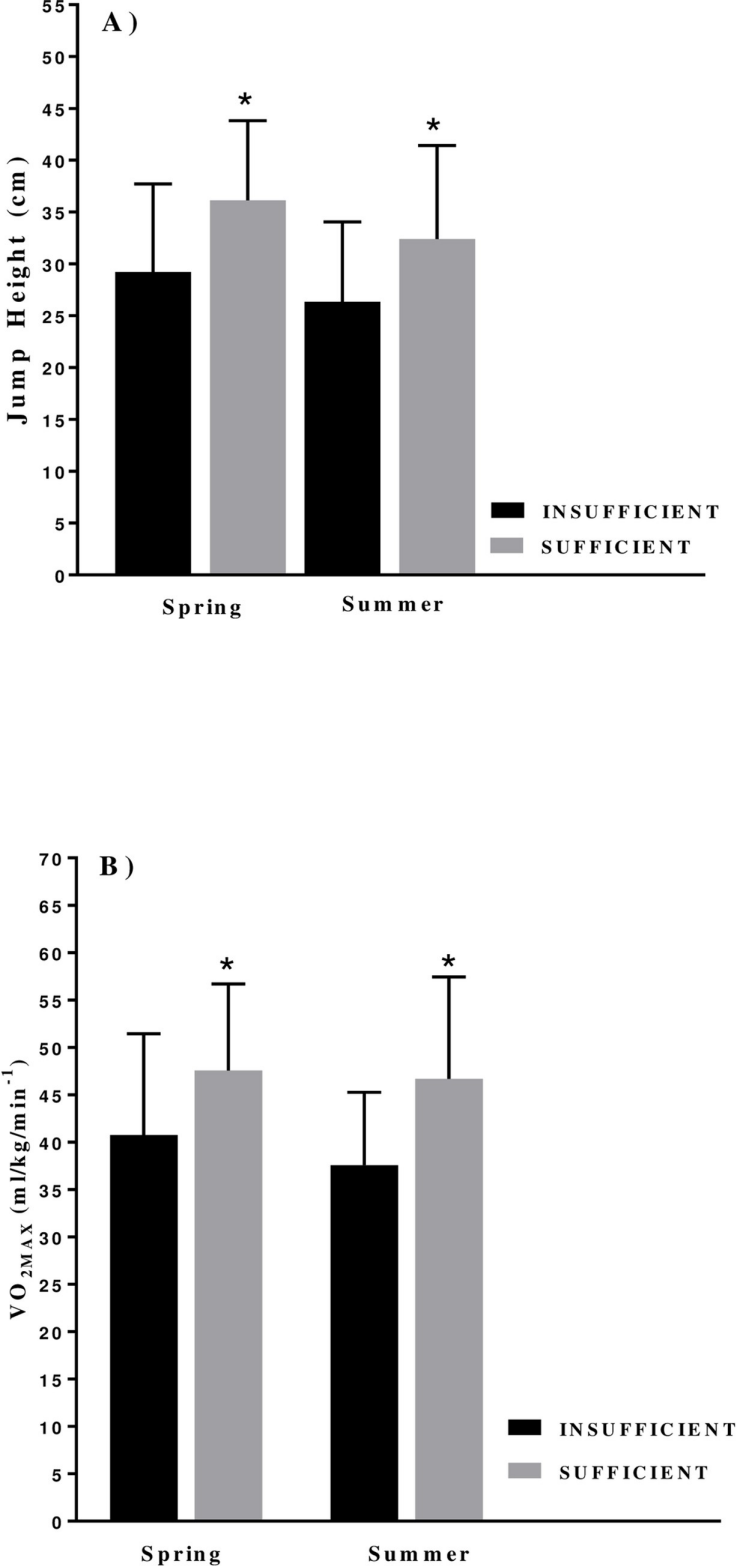
Mean (± SD) **A**) Jump height and **B**) Aerobic fitness (VO_2MAX_) within the insufficient (<50 nmol/L) and sufficient (>50 nmol/L) groups during the spring and summer term. *p = < 0.05.

Once separated into insufficient (<50 nmol/L [22]) and sufficient (>50 nmol/L [22]; Table 2) groups during both time points there was a distinct difference in performance parameters (Fig 3). The insufficient group (n = 31) had a lower CMJ height during spring when compared to the sufficient group (28.9 ± 8.7 vs. 36.1 ± 7.7cm; p = 0.055). However, following an ANCOVA test there was no significant difference in CMJ height when adjusted for sufficiency status, sporting group, gender, LBM, BF and age (*F* (1,11) 0.349, *p* = 0.6). The insufficient group had a lower aerobic fitness during the spring (40.8 ± 10.7 vs. 47.6 ± 9.1 ml/kg/min^-1^; p = 0.05), although it did not reach significance and was confounded by gender (p = 0.005), sport participation (p = 0.005), LBM (p = 0.035) and BF (p = 0.016). These findings were also mirrored in the summer term where the insufficient group’s (n = 12) CMJ height (26.4 ± 7.7 vs. 32.4 ± 9.0 cm; p = 0.05) and aerobic fitness (37.6 ± 7.7 vs. 46.7 ± 10.8 ml/kg/min^-1^; p = 0.012) were lower than their peers. Aerobic fitness was found to be confounded by gender (p = 0.03), sport participation (p = 0.01) and LBM (p = 0.004) after an ANCOVA test. CMJ height was also confounded by the same co-variates (*F* (1, 27) 0.046, *p* = 0.8). The predictor variables suggest that the male and athlete participants were likely to score higher than the female and control participants (*B* = +14.1 ml/kg/min^-1^and +12.8 ml/kg/min^-1^, respectively).

**Table 2:** Participant characteristics and physical performance parameters according to vitamin D status.

	Spring		Summer	
	Insufficient	Sufficient^1^	Insufficient	Sufficient^2^
**Male (n)**	15 (60%)	6 (25%)		
**Athletes (n)**	17 *(59%)*	12 *(86%)*	5 *(42%)*	26 *(79%)*
**Age (y)**	22.9 ± 3.5	21.3 ± 3.1	-	-
**BMI (kg/m^2^)**	23.5 ± 2.5	23.0 ± 1.7	23.0 ± 2.0	23.2 ± 2.0
**Body Fat (%)**	25.3 ± 7.6	20.5 ± 5.7*	28.1 ± 7.0	22.6 ± 7.6 ^α^
**LBM (kg)**	52.8 ± 12.1	56.6 ± 9.3	48.8 ± 12.1	54.2 ± 10.4
**Handgrip (kg)**	34.0 ± 9.5	34.4 ± 8.2	34.0 ± 13.9	39.1 ± 9.7
**KE (Nm)**	213.6 ± 71.5	215.1 ± 52.5	202.0 ± 72.1	240.7 ± 80.6
**CMJ (cm)**	27.2 ± 8.8	35.2 ± 7.1*	25.2 ± 7.7	31.6 ± 8.9^α^
**VO_2MAX_ (ml/kg/min^-1^)**	40.7 ± 10.9	47.2 ± 8.9	37.6 ± 7.7	46.7 ± 10.8^α^

Values mean ± SD.

‘Sufficiency’ level set at >50 nmol/L; ‘Insufficiency’ level set at < 50 nmol/L.

^1^ Independent t-test between insufficient and sufficient university students during the spring season*<0.05.

^2^ Independent t-test between insufficient and sufficient university students during the summer season ^α^<0.05.

## Discussion

The principle aim of this study was to examine the effects of regular physical exercise training on the relationship between vitamin D status and physical fitness in university athletes and sedentary controls. We found that in the spring term, 20% of our participants were deficient in vitamin D, according to the UK guidelines (<25nmol/L, [5]), half of which were athletes. We observed a significant improvement in vitamin D status during the summer in this non-supplemented population, which most likely can be explained by an increased exposure to UVB. An insufficient status (<50nmol/L) negatively impacted CMJ height across both seasons and groups, although when adjusting for other covariates this significant difference disappeared.

It is known that poor vitamin D status is common within Europe [23] and this has also been found specifically within the professional athletic community [2, 10]. Research on vitamin D status at varying latitudes at university sport level are predominantly reported in US populations [24–26]. To our knowledge, this is one of the first UK studies in a University population to explore the changes in vitamin D status from spring to summer through subcutaneous exposure and diet rather than supplementation in young adults. We found that 22% of the population were deficient (<25 nmol/L) during the spring, which is representative of the UK adult population as 29% of the population were found to be deficient in vitamin D from January to March by the National Diet and Nutrition Survey (NDNS) [27]. This can be attributed to the lack of UVB radiation available to produce vitamin D subcutaneously and the limited dietary vitamin D intake through the spring term (3.3 ± 2.7 μg/d). During the summertime, athletes are often encouraged to obtain plenty of safe sunlight exposure to further increase their vitamin D status combined with an adequate intake of food sources [28]. Our results show that university athletes and sedentary students alike improve their vitamin D status from spring to summer, which was not a result of the changes in dietary vitamin D intake. Indeed, all participants were able to increase vitamin D status, and none presented as deficient in the summer.

The vitamin D status of our student athletes at baseline (51.7 ± 20.5 nmol/L) was modest in comparison to younger athletes living at the same latitude in Poland (17.2 years; 51°N; 76 ± 37.3nmol/L) [29] but was similar to that reported in an Irish athlete cohort (55°N; 49.22nmol/L) in the spring [30]. Notably, the vitamin D status of our sedentary students were significantly lower during the spring (37.2 ± 18.9 nmol/L) and summertime (55.6 ± 18.8 nmol/L) when compared to our athletes and the aforementioned studies. Therefore, it could be suggested that sport participation and increased physical activity is conducive to maintaining a healthy vitamin D status throughout the year.

A limitation of our investigation is that sunlight exposure practices were not explored with a validated questionnaire, unlike other vitamin D studies [31]. Nonetheless we did measure direct sunlight exposure across the seasons with dosimetry. Our findings suggest that the university athletes had a higher subcutaneous exposure during the spring term (1.4 ± 1.1 SED) in comparison to the control group (1.4 ± 1.1 vs 0.8 ± 1.0 SED, respectively; p = 0.08). Notably, the control group presented with a higher fat mass during the spring (p = 0.003) and summer (p = 0.1). Therefore, as the average BMI for participants within the control group during both seasons were considered overweight (28.2 and 26.6 kg/m^2^, respectively) it could be suggested that an excess in adipose tissue within the control group contributed to lower 25(OH)D levels [5]. The control group also had a higher population of female participants (50%) when contrasted to the athlete group (44%).

Our results show that vitamin D insufficiency (<50nmol/L) was not associated with a lowered CMJ height in spring and summer, following adjustments for co-variates. This contrasts to previous literature regarding predictors of lower body muscular strength [32, 33] and countermovement jump [31, 32]. This could be attributed to our athletes having a lower baseline CMJ height 35.7 ± 6.8 when contrasted against the preliminary results from other cross-sectional studies: UK club-level athletes (47.0 ± 6.9cm [32]), south Korean collegiate athletes (54.1 ± 1.03cm [34]), polish ice-hockey players (42.4 ± 4.8cm [29]) and US collegiate athletes (47.21 ± 1.7cm [33]).

When evaluating our athletes’ lower body muscle strength we found that they presented with a peak KE of 241.6 ± 73.8 Nm at baseline, which improved to 251.3±103.2 Nm in the summer term following an observed improvement in vitamin D status. Despite this improvement, we found no statistically significant positive correlations between muscular strength and vitamin D status after controlling for co-variates such as gender, LBM, BF and age. This was also the case for other observational studies [17, 29, 31]. However, a recent systematic review with a meta-analysis found that there is a positive relationship between vitamin D supplementation and predictors of upper- or lower-body muscle strength. This was in agreement with another review assessing global muscular strength, although very small associations were found [33]. Therefore, our lack of findings could be attributed to a smaller cohort with a homogeneous vitamin D status.

Initially we observed a positive association between vitamin D sufficiency and aerobic fitness during the spring and summer (p = 0.05; p = 0.012, respectively) however, this disappeared following adjustments. Other cross-sectional studies found a positive correlation between vitamin D status and predictors of cardiovascular fitness within UK military recruits [16] and US students [17]. When controlling for co-variables no correlations have been found between vitamin D and aerobic capacity according to three recent observational studies investigating ice-hockey players [29, 35] and footballers [36]. Alike our study, the above-mentioned studies’ absence of correlations could be attributed to the smaller number of participants recruited (*n* < 50). Therefore, larger cohorts should control for co-variables adequately and determine the individual contribution of vitamin D on aerobic fitness.

As highlighted within the literature, a common limitation that confounds many meta-analysis investigating the effects of vitamin D upon exercise performance is large heterogeneity [11, 33, 37]. This is due to many studies utilising different methods of measuring 25(OH)D status, such as ELISA assays rather than the gold standard; LC-MS. Studies also use a large variety of predictors for aerobic capacity and muscular strength or power. This includes VO_2max_ tests [30], 1.5-mile endurance time trials [16], 10/20M sprints [32], shuttle runs [34], repeated sprint ability [29], 1 maximal repetition measurements [32], isokinetic dynamometry [36] and vertical jump height [31]. Moreover, some studies have a large variety of ethnicities in their recruits [36] which can be a confounding factor due to the inhibitory nature of melanin [4]. Thus, it is suggested by the authors that future research focuses upon well-designed randomised controlled trials (RCTs) with adjustments for confounding factors. Although this was not an RCT, the authors did control for ethnicity and sunlight exposure.

The secondary aim of this study was to assess the dietary vitamin D intake of UK university students. As highlighted by the SACN [5], there is limited evidence on the vitamin D status and intake of healthy young adults across the seasons. Our study showed that athletes had a lower vitamin D intake when contrasted to controls during the spring (3.0 ± 2.5 vs. 3.9 ± 2.9μg/d) and summer (2.7 ± 2.3 vs. 5.9 ± 4.8 μg/d). Which, could be attributed to the smaller group of controls recruited within this study. This contradicts a study conducted within a US group (64°N) [31], where their sedentary male controls had a significantly lower vitamin D intake than their male collegiate athletes (8.7 ± 4.4 vs. 1.8 ± 0.7 μg/d) and did not meet the US recommended dietary allowances (RDA; 15 μg/d). This was also replicated in another US study (41°N), where only 4% of collegiate athletes in the spring term met their RDA [24]. Our findings are representative of a UK population and are in line with the NDNS, with men and women on average consuming 3.9 and 3.4 μg/d, respectively [38]. Notably, alike the US studies, neither group met the UK RNI (10 μg/d) [5]. Overall, this study contributes to the current knowledge regarding the vitamin D status, dietary intake and sunlight exposure of a variety of free-living university athletes over two academic semesters at a higher latitude (51°N). This information could potentially be beneficial to inform the risk of vitamin D insufficiency amongst young adult populations.

## Conclusions

In conclusion, predictors of physical performance were not associated with vitamin D status within both groups or during both seasons. We also provide information on the vitamin D intake of healthy young adults, for which there is limited evidence. The novelty of this study was that it explored the physical fitness of University level athletes and sedentary students within the UK. As University sport within the UK is becoming more competitive and participation continuously increases annually it is relevant that vitamin D within this cohort be assessed. This is because current research from US collegiate groups are not comparative to our cohort. Overall, these data suggest that University athletes and students are at risk of vitamin D deficiency during the winter and further research is warranted on the interrelationship between low vitamin D status, athletic performance and overall health for those residing at northern latitudes, such as the UK.

## Supporting information

S1 Questionnaire Health screening questionnaire. (PDF)

S2 Questionnaire UoS DEXA questionnaire. (PDF)
