# Supplementary material for: The relationship between vitamin D status, intake and exercise performance in UK University-level athletes and healthy inactive controls
Source: PLoS One. 2021 Apr 2;16(4):e0249671. doi: 10.1371/journal.pone.0249671 (PMC8018647; doi:10.1371/journal.pone.0249671)
Supplement: S1 Questionnaire — (PDF) [file pone.0249671.s001.pdf]

THE D-BIICEP STUDY: HEALTH & LIFESTYLE SCREENING QUESTIONNAIRE

The purpose of this questionnaire is to assess whether you are suitable to take part in the study and that it is safe for you to do so. Please answer the questions as honestly and accurately as you can and remember there are no right or wrong answers to the questions. Your answers will be kept completely confidential.

Name: **Date of birth**: **Age:**

Address:

Contact telephone number:

Gender: M / F

Height: ___________ cm **Weight**: **____________ kg**

Ethnicity (please select):

Ethnic group		Please tick
**White**	British	
	Irish	
	Any other White background (please state)	
**Asian or Asian British**	Indian	
	Pakistani	
	Bangladeshi	
	Any other Asian Background (please state)	
**Arab or Arab British**	Kuwait	
	Bahrain	
	Qatar	
	United Arab Emirates	
	Oman	
	Yemen	
	Saudi Arabia	
	Any other Arab Background (please state)	

1. **Do you wear a veil or any form of head dress?** **YES ☐ NO ☐**
2. **Were you born in the UK?** **YES ☐ NO ☐**

   (If no, please specify where)……………………………………….

3. Initial Considerations

Do you have a muscle/ joint problem that is aggravated by exercise? **YES** ☐ **NO** ☐

Are you feeling unwell today, or had a fever in the last 7 days? **YES** ☐ **NO** ☐

4. Habitual Physical Activity

Do you perform moderate exercise regularly (at least twice a week)? **YES** ☐ **NO** ☐

Do you perform vigorous exercise regularly (at least twice a week)? **YES** ☐ **NO** ☐

Which Team Surrey sport are you involved in?

…………………………………………………………………………………………….

What level of competition do you perform at?

University League (BUCS) ☐

County ☐

National ☐

International ☐

How long have you been competing at this level?

………………………. (years)

How many hours a week do you train/ compete in your sport?

Train: ……………………………………………………..

Compete: …………………………………………………..

How many hours a week do you perform the following training modes?

Aerobic/ endurance: ………………….

Resistance:……………………….

Sport specific play/ practice ………………………..

Other…………………………………………………..

How many hours a week do you train and compete outside? Please indicate if there is a seasonal variation to your response.

Autumn/ Winter……………………….

Spring/ Summer ……………………..

5. Competition and Training History

How long have you been training for your chosen sport?

……………………. (years/ months)

How long have you been competing in your chosen sport?

……………………. (years/ months)

Please indicate if there are any other activities/ sports you have participated in over the past 10 years. For each activity you identify please consider the time period of participation (years, months/ year, hours/ month etc).

……………………………………………………………………………………………

During years 14-18, how often did you regularly participate in sport and leisure physical activity, excluding walking?

0-1 hours/week ☐

2-3 hours/ week ☐

4-7 hours/ week ☐

Over 7 hours/ week ☐

During years 14-18, how many miles did you normally walk each day?

Under 1 mile ☐

1-2 miles ☐

3-5 miles ☐

Greater than 5 miles ☐

6. **Have you ever had any of the following:**

	YES	NO
Prior/ present history of coronary heart disease, angina, heart attack or stroke		
Prior/present history of Type 1 or Type 2 Diabetes.		
Prior/present history of Thyroid disease		
Prior/present history of osteoporosis, osteopenia or a musculoskeletal disease		
Prior/present history of a blood disorder (except mild anaemia)		
Prior/present history of malignancy		
Prior/present history of a digestive problem		
Prior/present history of liver or kidney disease.		
Prior/present history of clinical depression or other psychological disorders.		
Prior/present history of eating disorders.		
Prior/present history of drug or alcohol abuse within the last 2 years.		
Suffered from a head injury		
Prior/ present disturbance of vision/ balance/ coordination		

7. **Signs and Symptoms.**
   Do you experience any of the following**:**

	YES	NO
Chest discomfort with exertion		
Shortness of breath at rest or with mild exertion		
Feeling faint or have spells of severe dizziness		
Swelling or a build-up of fluid in or around your ankles		
Difficulty with breathing when lying down		
The feeling your heart is racing/ skipping beats at rest or during exercise		
Pain in your lower legs during exercise, not due to soreness or stiffness		
Unusual fatigue or shortness of breath during everyday activities		
Has your doctor ever told you that you have a heart murmur		

8. **Medication and Allergy Information.**

Do you have any allergies or food intolerances? **YES ☐ NO ☐**

Are you allergic to any medication? **YES ☐ NO ☐**

Are you allergic to plasters? **YES ☐ NO ☐**

If yes to any of the above, please provide additional information as to which food or medication you are allergic to:

……………………………………………………………………………………………

……………………………………………………………………………………………

Are you on any prescribed medication? **YES ☐ NO ☐**

If yes, please specify what type of medication this is and how often you take the medication:

……………………………………………………………………………………………

……………………………………………………………………………………………

Do you take any vitamin supplements containing vitamin D?

YES ☐ NO ☐ DON'T KNOW ☐

If yes please specify:

- How many months you have been taking the supplements……………
- The dose of the supplements (if known)…………………………………
- The brand of the supplements (if known) ………………………………..

9. **Are you currently on a weight-reducing diet or other dietary restrictions (except vegetarianism)?**

YES ☐ NO ☐

If yes, please provide details:

……………………………………………………………………………………………

……………………………………………………………………………………………

10. **Risk Factors.**

Do you currently smoke/ have quit within the previous 6 months? YES ☐ NO ☐

If yes, please specify how many per day ……………………………………..............

Do you drink alcohol? **YES ☐ NO ☐**

If yes, please specify how many units per week ……………………………………………

(please see below for a guide on number of units of alcohol in common drinks)

Alcohol	Measure	Unit
Ordinary strength lager (4%) *(e.g. Carling, Fosters)*	Pint	2.3
Strong lager (5.2%) *(e.g. Stella Artois, Kronenburg)*	Pint	3
Strong lager *(e.g. Stella Artois, Carlsberg Export, Grolsch)*	440ml can	2.2
Beer/ordinary strength Ale *(e.g. John Smith's, Guinness)*	Pint	2.3
Red/White Wine	Std 175ml	2
Red/White Wine	Lg. 250ml	3
Spirits	Std 25ml	1
Spirits	Lg. 35ml	1.4
Alcopop e.g. Smirnoff Ice, Bacardi Breezer, VK	275ml	1.5

Do you have high blood pressure? **YES ☐ NO ☐**

Has your doctor ever told you that you have high cholesterol? **YES ☐ NO ☐**

Is there a history of Heart Disease or Stroke in your family? **YES ☐ NO ☐**

11. Have you been abroad or on holiday during the past 12 months? **YES ☐ NO ☐**

If yes, please specify where this was and the month this holiday was taken:

Country of visit…………………………………………………………..……..

Month/year…………………………………………………………………..

Country of visit…………………………………………………………...…..

Month/year…………………………………………………………………..

12. Are you planning any holidays abroad during the next 12 months? **YES ☐ NO ☐**

If yes, please specify where and when this holiday will be taken:

Country of visit…………………………………………………………...…..

Month/year…………………………………………………………………..

Country of visit…………………………………………………………...…..

Month/year…………………………………………………………………..

13. When you are out in the sun do you wear sunscreen (at any time of the year)?

YES ☐ NO ☐

If yes, specify the factor of sunscreen used when at home (in the UK) and on holiday:

At home………………………………………………………………………

On holiday (can include beach/ skiing holidays)…………………………

14. Do you use sunbeds? **YES ☐ NO ☐**

If yes, please state how often you use them:

Once a week ☐

Once a month ☐

More than 6 times per year ☐

Less than 6 times per year ☐

Occasionally ☐

For female participants only:

Are you currently pregnant or planning a pregnancy during the next 12 months?

YES ☐ **NO ☐**

Please provide contact details of a suitable person for us to contact in the event of any incident or emergency.

Name:……………………………………………………………………………………………

Telephone number:…………………………………………………………………………….

Work ☐ Home ☐ Mobile ☐

Relationship to participant…………………………………………………………………..

Are you currently involved in any other research studies at the University or elsewhere?

YES ☐ **NO ☐**

If yes, please provide details of the study……………………………………………………..

Thank you for taking the time to complete this questionnaire.

Does participant meet inclusion/exclusion criteria?

YES ☐

Baseline visit: Date ……………………..…….. Time…………………….

Participant number assigned………………………………………..

NO ☐

Reason: ………………………………………………………………

………………………………………………………………………

………………………………………………………………………

Questionnaire completed by:

Study Investigator: …………………...…………………..   Date ………………………..

(Print name)
