# Supplementary material for: The relationship between vitamin D status, intake and exercise performance in UK University-level athletes and healthy inactive controls
Source: PLoS One. 2021 Apr 2;16(4):e0249671. doi: 10.1371/journal.pone.0249671 (PMC8018647; doi:10.1371/journal.pone.0249671)
Supplement: S2 Questionnaire — (PDF) [file pone.0249671.s002.pdf]

UNIVERSITY OF SURREY

Sport & Exercise Science Laboratories

DEXA Questionnaire

Please complete the following questions to the best of your ability. If you would like more information or have any questions, please ask the DEXA operator for assistance.

1. Have you had any medical investigations, or treatments involving x-rays or radiation within the last week? ☐ *Yes* ☐ *No*

   Some examples of investigations using x-rays include; CT scans, dental x-rays, scans for suspected broken bones, radiotherapy, mammograms, other bone or body scans

   If 'Yes', please give details ……………..…………………………..…………………………

   …………………………………………………………………………………………………....

2. Have you had any medical investigations, or treatments involving x-rays within the last month? ☐ *Yes* ☐ *No*

   If 'Yes', please give details ……………..…………………………..…………………………

   …………………………………………………………………………………………………....

3. Have you had any medical investigations, or treatments involving x-rays within the last year? ☐ *Yes* ☐ *No*

   If 'Yes', please give details ……………..…………………………..…………………………

   …………………………………………………………………………………………………....

4. Do you have any of the following medical devices in, or on your body?

Please tick any which apply & describe the device in the adjacent space provided.

a. ☐ Ostomy devices (e.g. colostomy bag) ……………………………………..
b. ☐ Prosthetic devices (e.g. hip or knee replacement) ………………….……….
c. ☐ Surgical devices (e.g. metal clips, pins, plates) ……………………..………
d. ☐ Pacemaker or pacemaker leads ....
e. ☐ Radioactive implants (i.e. for treatment of cancer) ………………………….
f. ☐ Catheters or tubes ……………………..………………………………………

5. Are you exposed to radiation as part of your job? ☐ *Yes* ☐ *No*

Some occupations that may be exposed to radiation include; aircraft pilot, flight attendant, scientist, worker at a nuclear power station

6. Do your clothes contain any metal such as buttons, zips, or clips? ☐ *Yes* ☐ *No*

Any clothes containing metal will need to be removed before you have the DEXA scan. Tops and trousers are available, in the changing room, to change into, and lockers are provided.

7. Are you wearing any jewellery? ☐ *Yes* ☐ *No*

Any jewellery, including watches and piercings will need to be removed before you have the DEXA scan. If you are unable to remove any items, please inform the DEXA operator.

8. Do you have any foreign objects in your body? ☐ *Yes* ☐ *No*

e.g. shrapnel, buckshot, metal of any kind

For females only;

9. Is there any possibility that you might be pregnant? ☐ *Yes* ☐ *No*

The DEXA scan involves you receiving a low dose x-ray. It is not advisable for you to have this scan if you are pregnant. Pregnancy test kits (requiring a urine sample) are available in the toilets if you would like to check whether you are pregnant. Please speak with the DEXA operator if you do not wish to proceed with the scan, but you do not need to disclose the reason why.

I certify that the information given above is, to the best of my knowledge, true and correct.

Name: ……………………………………………………….

Signed: …………………………………………………….. Date: ……/……../………

Witnessed:

Name: ……………………………………………………….

Signed: …………………………………………………….. Date: ……/……../………
